# Tropomyosin isoforms have specific effects on the transcriptome of undifferentiated and differentiated B35 neuroblastoma cells

**DOI:** 10.1002/2211-5463.12386

**Published:** 2018-02-19

**Authors:** Holly Stefen, Alexandra Kalyna Suchowerska, Bei Jun Chen, Merryn Brettle, Jennifer Kuschelewski, Peter William Gunning, Michael Janitz, Thomas Fath

**Affiliations:** ^1^ Neurodegenerative and Repair Unit School of Medical Science UNSW Sydney NSW Australia; ^2^ School of Biotechnology and Biomolecular Sciences UNSW Sydney NSW Australia; ^3^ Cellular and Genetic Medicine Unit School of Medical Sciences UNSW Sydney NSW Australia

**Keywords:** actin cytoskeleton, RNA‐seq, tropomyosin isoforms

## Abstract

Tropomyosins, a family of actin‐associated proteins, bestow actin filaments with distinct biochemical and physical properties which are important for determining cell shape and regulating many cellular processes in eukaryotic cells. Here, we used RNA‐seq to investigate the effect of four tropomyosin isoforms on gene expression in undifferentiated and differentiated rat B35 neuroblastoma cells. In undifferentiated cells, overexpression of tropomyosin isoforms Tpm1.12, Tpm2.1, Tpm3.1, and Tpm4.2 differentially regulates a vast number of genes, clustering into several gene ontology terms. In differentiated cells, tropomyosin overexpression exerts a much weaker influence on overall gene expression. Our findings are particularly compelling because they demonstrate that tropomyosin‐dependent changes are attenuated once the cells are induced to follow a defined path of differentiation.

**Database:**

Sequence data for public availability are deposited in the European Nucleotide Archive under the accession number PRJEB24136.

**Abbreviations**
ADF: actin‐depolymerizing factor
ADP: adenosine diphosphate
ATP: adenosine triphosphate
cAMP: cyclic adenosine monophosphate
DEG: differentially expressed gene
DNA: deoxyribonucleic acid
FPKM: fragments per kilobase of transcript per million mapped reads
FRAP: fluorescence recovery after photobleaching
GFP: green fluorescence protein
GO: gene ontology
RNA: ribonucleic acid
RNA‐Seq: RNA sequencing
sfGFP: superfold GFP
Tpm: tropomyosin

The actin cytoskeleton provides structural support and is essential for the cellular morphogenesis of eukaryotic cells and many physiological processes including cell motility, endocytosis, transport of organelles, apoptosis, and the maintenance of correct directional signal transmission in neurons 1, 2. Several actin‐binding proteins are known to bestow the actin cytoskeleton with a high degree of structural and functional diversity by regulating actin filament turnover, filament branching, bundling, and cross‐linking of individual filaments 3. Tropomyosins (Tpms) are a large family of actin‐binding proteins that are generated by alternative splicing from four different genes (*Tpm1*,* Tpm2*,* Tpm3,* and *Tpm4*). They not only define distinct filamentous actin populations in different cell populations and subcellular compartments, but also regulate the access of other actin‐binding proteins to the actin filament 4, 5. Tpms have been identified to play an important role in a range of cellular processes with changes in protein expression, including the regulation of cell transformation 6, 7, ERK‐mediated proliferation 8, insulin‐stimulated GLUT4 transport 9, and anoikis 10. The regulation of these diverse cellular processes suggests that an altered composition of Tpm expression in eukaryotic cells will lead to changes in cellular pathways at a global level rather than impacting on interactions with only a select number of established interaction partners. In this study, we aimed to test whether the level of expression of Tpms determines the cell transcriptome in an isoform‐specific manner and whether these changes are dependent on the differentiation stage of these cells. Rat B35 neuroblastoma cells 11, 12 have extensively been used to study cellular processes of eukaryotic cells, including neuronal morphogenesis 11, 13, cell motility 14, 15, vesicular trafficking 16, and apoptosis 17, 18. Our group has used the B35 cell system previously to study the role of different Tpm isoforms in neuronal cell morphogenesis 13, 19. Stable rat B35 neuroblastoma cell lines overexpressing the Tpm isoforms Tpm1.12, Tpm2.1, Tpm3.1, and Tpm4.2, from each of the Tpm genes 1‐4, were previously generated 13, 17, 19. Neuronal differentiation requires the coordinated reorganization of the actin cytoskeleton to facilitate the sprouting and elongation of neurites, which ultimately form axons and dendrites. Our previous studies showed that the overexpression of Tpms in B35 cells was not only sufficient to induce the formation of neurites, but also differentially influenced neurite branching and extension in differentiating B35 cells 13.

In this study, we wanted to understand how the transcriptome of B35 cells is altered in response to the overexpression of different Tpm isoforms. The analysis of the transcriptome does not only provide insight into which genes are being expressed, and at what level, but also sheds light onto how the distinct expression of genes could alter essential cellular pathways and mechanisms of cellular morphogenesis.

We employed Illumina RNA‐seq which has been demonstrated to yield higher sensitivity, deeper resolution, and greater reproducibility when compared to conventional genomic methods such as microarray analysis 20. Another advantage of RNA‐seq is the ability to identify novel transcripts and splice variants, which is not possible using microarray analysis. To identify gene clusters of biological pathways, which may reveal hidden patterns that regulate specific biological processes, differentially expressed genes (DEGs) were further analyzed via clusterProfiler. The main advantage of clusterProfiler is the application of both biological term classification and enrichment analysis to gene cluster analysis, thereby providing greater insight into understanding higher order functions in biological systems 21. We found a large number of changes in genes in undifferentiated B35 cells, compared with differentiated cells, where Tpm overexpression appeared to have less influence on gene expression. Differentially expressed genes (DEGs) found in undifferentiated cells could be grouped into a range of different pathways, including pronounced changes in actin‐binding pathways. However, in differentiated cells, only one isoform, Tpm3.1, had DEGs that generated gene ontology (GO) terms, suggesting limited pathway commonality in differentiated Tpm1.12, Tpm2.1, and Tpm4.2 B35 cells. Our results are consistent with the overall hypothesis that different Tpm isoforms generate distinct actin filament populations that are controlling key pathways of cellular function in eukaryotic cells.

## Materials and methods

### Cell culture, differentiation, and harvesting

B35 rat neuroblastoma cells, stably overexpression of different Tpm isoforms, were previously described for the overexpression of Tpm1.12 and Tpm3.1 19, Tpm2.1 17, and Tpm4.2 13. Cells were cultured in Dulbecco's modified Eagle's medium (DMEM; Invitrogen, Life Technologies, Melbourne, Vic., Australia), 0.6% geneticin (Invitrogen, Life Technologies), and 10% heat‐inactivated fetal bovine serum (FBS; Invitrogen, Life Technologies) at 37 °C in a 5% CO_2_ incubator. To differentiate the cells, media was changed to DMEM containing 0.1% FBS, 0.5 mm cyclic adenosine monophosphate (cAMP) 24 h prior to harvesting. Cells were harvested by incubation with 1% trypsin in phosphate‐buffered saline and pelleting of the cells via centrifugation at 300 g for 10 min. The pellets were snap‐frozen in liquid nitrogen and stored at −80 °C until RNA sequencing.

### RNA preparation and sequencing

Total RNA was isolated from three biological replicates from each transgenic cell line and empty vector control‐transfected cells, using RNeasy Mini Kit (Qiagen, Hilden, Germany) followed by RNase‐free DNase treatment to remove traces of genomic DNA. The Agilent 2100 Bioanalyzer RNA Nano Chip was used to assess the RNA quality of the total RNA. The RNA integrity number (RIN) values ranged between 6.0 and 7.0. RNA was generated using Illumina TruSeq RNA sample preparation for poly(A) RNA and sequenced using paired‐end 100‐bp reads on Illumina HiSeq2500.

### Read mapping, transcript assembly, and comparative analysis

Sequencing files in FASTQ format were uploaded to Galaxy server at http://usegalaxy.org sequence reads mapped to the rat reference genome (Rnor_6.0) using TopHat (version 2.1.1) as described previously 22. The BAM files from TopHat were then fed into Cufflinks (version: 2.2.1) on Galaxy server for transcript assembly and expression level calculation. Annotation files from Ensembl rat genome assembly Rnor_6.0 were used as reference annotation. Next, Cufflinks‐assembled transcripts were merged together using Cuffmerge on Galaxy server using uploaded reference annotation. Differential expression analysis was performed using Cuffdiff. Cuffdiff utilizes the merged files from Cuffmerge along with the original alignment files produced from TopHat to calculate expression levels and determine their statistical significance and whether the transcripts are differentially expressed. Genes with an FPKM ≥ 1 in at least one condition were considered as expressed.

### Pathway analysis

The ClusterProfiler program 21 was used through the platform R studio (https://www.rstudio.com/) version 1.0.143 to undertake the pathway analysis of the significant differentially expressed linear RNA. Pathway analysis included matching the input list of annotated genes expressing linear RNA to their ENTREZ ID. These genes were then matched to their respective GO terms, and an over‐representation test, based on hypergeometric distribution of the GO terms, was performed to identify enriched GO terms and subsequently measure the statistical significance of each enriched GO term. For this pathway analysis, the allocated ontology term option used was biological processes. The scripts used for the pathway analysis were obtained from https://bioconductor.org/packages/devel/bioc/vignettes/clusterProfiler/inst/doc/clusterProfiler.html.

## Results and Discussion

### Overexpression of tropomyosin isoforms differentially affects gene expression in undifferentiated and differentiated B35 neuroblastoma cells

**Figure 1:**
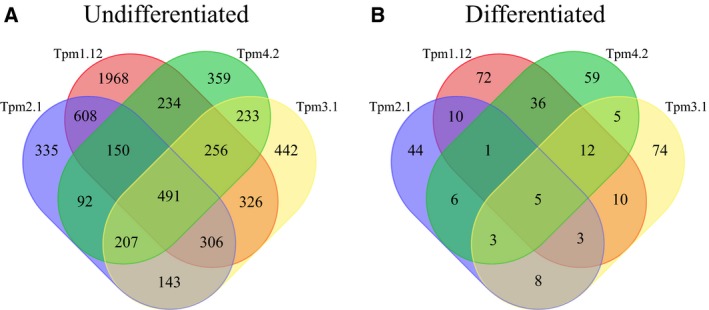
Overexpression of Tpm isoforms differentially regulates gene expression in undifferentiated and differentiated rat B35 neuroblastoma cells. (A) In undifferentiated cells, overexpression of tropomyosin isoforms 1.12, 2.1, 3.1, and 4.2 differentially regulates a large number of genes with partial overlap between isoforms. (B) Once differentiated, Tpm isoforms have a weaker influence on gene expression and less overlap between isoforms compared with undifferentiated cells.

In undifferentiated cells, the overexpression of specific Tpm isoforms alters the expression of thousands of genes, with a surprising degree of difference in effected genes between isoforms (Fig. 1A). Cells with increased levels of Tpm1.12 had changes in the expression of over 4000 genes, the highest number of observed DEGs out of the four isoforms investigated. Approximately 45% of these DEGs were specific to Tpm1.12‐overexpressing cells. Tpm2.1, Tpm3.1 and Tpm4.2 each had between 2000 and 2400 DEGs with isoform specificity ranging between 15 and 20%.

In differentiated cells, numbers of DEGs were overall greatly reduced compared with undifferentiated cells (Fig. 1B). The highest number of DEGs observed was 160 and again associated with Tpm1.12 overexpression. Differentiated cells, overexpressing Tpm3.1 and Tpm4.2, had similar numbers of DEGs, 120 and 136, respectively. Tpm2.1 had, with 89, the fewest number of DEGs. In terms of specificity, once differentiated, there was also reduced overlap between isoforms. Tpm3.1 showed relatively high specificity, with over 60% of observed DEGs being unique to cells, overexpressing this isoform. Tpm2.1 and Tpm4.2 isoforms had specificity ranging between 49% and 43%, respectively, much greater than observed in undifferentiated cells. Tpm1.12 was the only isoform that showed similar specificity of approximately 45%, regardless of whether the cells were differentiated or not. Overall, these results indicate that Tpms exert a broad influence on gene expression in undifferentiated cells. However, once cells undergo differentiation, gene expression appears more tightly regulated with tropomyosin having a weaker but more isoform‐specific influence.

### Undifferentiated Tpm1.12‐, Tpm3.1‐, and Tpm4.2‐overexpressing B35 cells display some similarity in gene regulation

In undifferentiated cells, there was some commonality between the different cell lines (Table 1). In undifferentiated Tpm1.12‐overexpressing cells, *Ntm,* which encodes for a protein that inhibits neurite outgrowth 23, was the most downregulated gene. Within undifferentiated Tpm2.1 cells, *Bnc1* was among the top upregulated genes. Bnc1 is a transcription factor that plays a role in the expression of genes involved in cellular differentiation and proliferation 24. In Tpm3.1‐ and Tpm4.2‐overexpressing B35 cells, the gene encoding for neurofilament light chain, *Nefl*, was among the top downregulated genes, suggesting cross‐talk between the actin and intermediate filament systems. Together, this indicates the greatest commonality between Tpm3.1‐ and Tpm4.2‐overexpressing cells, both in the undifferentiated and in differentiated states. The similarity in effects of Tpm3.1 and Tpm4.2 has also been observed in other systems. *In vitro* single filament assays have shown that Tpm3.1 and Tpm4.2 often localize to the same F‐actin populations and have similar, rapid association with less cooperative binding to F‐actin 25. This was analyzed using FRAP experiments where the fluorescence recovery of sfGFP (super‐folder GFP) fusions of Tpm3.1 and Tpm4.2 was much more rapid, when compared to higher molecular weight Tpm isoforms. These isoforms also stimulate the ATPase activity of nonmuscle myosin IIa and, *in vitro*, do not efficiently protect filaments from the severing action of ADF/cofilin 25.

**Table 1:** Top 10 up‐ and downregulated genes in undifferentiated B35 neuroblastoma cells overexpressing tropomyosin isoforms Tpm1.12, Tpm2.1, Tpm3.1, and Tpm4.2

Gene	Locus	FPKM_Tpm1.12	FPKM_WT	Fold change	*P*_value	*q*_value	Gene_id	Gene	Locus	FPKM_Tpm1.12	FPKM_WT	Fold change	*P*_value	*q*_value	Gene_id
Tpm1.12 undiff. top 10 downregulated								Tpm1.12 undiff. Top 10 upregulated							
Ntm	chr8	4.5128	0.224026	−4.33228	5.00E‐05	0.00040997	XLOC_019559	–	chr2	0.330884	10.2837	4.95789	5.00E‐05	0.00040997	XLOC_011049
Krt15	chr10	288.734	14.6227	−4.30346	5.00E‐05	0.00040997	XLOC_004359	Rab15	chr6	0.0990585	3.02151	4.93084	0.0002	0.0014157	XLOC_017230
Chrdl1	chrX:	1.73954	0.0968726	−4.16647	5.00E‐05	0.00040997	XLOC_021556	–	chr15	0.0684066	1.87164	4.77403	0.0002	0.0014157	XLOC_007916
RGD1563159	chr18	3.41646	0.21943	−3.96067	5.00E‐05	0.00040997	XLOC_009428	Klf12	chr15	0.0662939	1.5938	4.58745	5.00E‐05	0.00040997	XLOC_007841
Kit	chr14	2.38455	0.17741	−3.74856	5.00E‐05	0.00040997	XLOC_007027	–	chr12	0.598639	13.742	4.52077	5.00E‐05	0.00040997	XLOC_005277
Rn18s, Rn45s, Rn5‐8s	chr14	2921.03	236.372	−3.62735	5.00E‐05	0.00040997	XLOC_006757	Kdf1	chr5	0.0728308	1.66214	4.51235	0.00055	0.00339959	XLOC_015675
Clec2 dl1	chr4	16.5433	1.44599	−3.51611	5.00E‐05	0.00040997	XLOC_014476	–	chr3	0.313492	6.41526	4.35501	5.00E‐05	0.00040997	XLOC_012148
–	chr3	5.1911	0.512821	−3.33951	0.00175	0.00899966	XLOC_012887	Spp1	chr14	0.0841515	1.66596	4.30722	0.0026	0.0124381	XLOC_006940
Ptpn7	chr13	14.9719	1.481	−3.33761	5.00E‐05	0.00040997	XLOC_006040	Spon1	chr1	0.205599	3.44618	4.06709	5.00E‐05	0.00040997	XLOC_000788
Hs3st6	chr10	2.46299	0.248645	−3.30825	0.0028	0.0132381	XLOC_003048	Igf2 bp1	chr10	0.257626	3.9438	3.93624	5.00E‐05	0.00040997	XLOC_004304

Gene	Locus	FPKM_Tpm2.1	FPKM_WT	Fold change	*P*_value	*q*_value	Gene_id	Gene	Locus	FPKM_Tpm1.12	FPKM_WT	Fold change	*P*_value	*q*_value	Gene_id
Tpm2.1 undiff. top 10 downregulated								Tpm2.1 undiff. Top 10 up‐regulated							
Pcdh9	chr15	74.6347	1.24766	−5.90255	5.00E‐05	0.00040997	XLOC_007836	Rab15	chr6:	0.068894	3.02151	5.45475	0.004	0.0177903	XLOC_017230
Aqp8	chr1	24.1952	1.10004	−4.4591	5.00E‐05	0.00040997	XLOC_000830	Pdpn	chr5	1.13354	13.7824	3.60392	5.00E‐05	0.00040997	XLOC_016368
Ntm	chr8	3.29835	0.224026	−3.88001	5.00E‐05	0.00040997	XLOC_019559	Clec12a	chr4	0.366014	3.56542	3.2841	0.00125	0.00679032	XLOC_014481
Cnrip1	chr14	1.60263	0.145715	−3.45922	0.01095	0.0400834	XLOC_006874	Qprt	chr1	0.761786	6.36273	3.06219	5.00E‐05	0.00040997	XLOC_002237
Ptpn7	chr13	13.9013	1.481	−3.23057	5.00E‐05	0.00040997	XLOC_006040	–	chr11	0.358316	2.71438	2.92132	0.0014	0.00745855	XLOC_004806
–	chr7	4.78368	0.511835	−3.22437	5.00E‐05	0.00040997	XLOC_018001	Bnc1	chr1	0.87852	6.59516	2.90826	5.00E‐05	0.00040997	XLOC_001936
–	chr5	6.86562	0.755452	−3.18398	0.01145	0.0415397	XLOC_016457	–	chr2	0.64662	4.39964	2.76639	0.0031	0.0144126	XLOC_011435
Dusp27	chr13	3.3696	0.451684	−2.89919	5.00E‐05	0.00040997	XLOC_006430	–	chr5	0.846829	5.75961	2.76583	5.00E‐05	0.00040997	XLOC_015850
–	chr6	27.8064	3.923	−2.82539	0.00715	0.0285097	XLOC_017447	Cpq	chr7	0.209266	1.4142	2.75658	0.0016	0.00835597	XLOC_017738
Socs2	chr7	5.24415	0.757575	−2.79125	5.00E‐05	0.00040997	XLOC_018257	Vsig10	chr12	0.423961	2.77583	2.71092	5.00E‐05	0.00040997	XLOC_005799

Gene	Locus	FPKM_Tpm3.1	FPKM_WT	Fold change	*P*_value	*q*_value	Gene_id	Gene	Locus	FPKM_Tpm1.12	FPKM_WT	Fold change	*P*_value	*q*_value	Gene_id
Tpm3.1 undiff. top 10 downregulated								Tpm3.1 undiff. Top 10 up‐regulated							
–	chr10	3.14938	0.088868	−5.14726	0.0002	0.0014157	XLOC_004626	Gabra1	chr10	0.970416	32.1676	5.05086	5.00E‐05	0.00040997	XLOC_003914
Kcnn4	chr1	22.3992	0.757394	−4.88626	5.00E‐05	0.00040997	XLOC_000310	–	chr5	0.168244	3.14325	4.22363	0.0002	0.0014157	XLOC_016486
Nefl	chr15	47.6865	1.67599	−4.8305	5.00E‐05	0.00040997	XLOC_007467	Nfia	chr5	0.107033	1.64896	3.94543	0.0033	0.0151739	XLOC_015473
–	chr19	10.7555	0.456691	−4.55771	5.00E‐05	0.00040997	XLOC_010104	Ndn	chr1	0.780198	8.50889	3.44706	5.00E‐05	0.00040997	XLOC_000525
–	chr15	2.9081	0.143898	−4.33695	0.00095	0.00540022	XLOC_007901	RGD1312005	chr18	0.283849	2.89758	3.35165	5.00E‐05	0.00040997	XLOC_009262
–	chr1:	1.66185	0.088369	−4.23311	0.00225	0.0110774	XLOC_002911	Ptprk	chr1	0.13888	1.38178	3.31462	0.00165	0.00857402	XLOC_001371
–	chr12	2.22161	0.119614	−4.21515	5.00E‐05	0.00040997	XLOC_005896	Klf12	chr15	0.175186	1.5938	3.1855	5.00E‐05	0.00040997	XLOC_007841
–	chr7	2.66362	0.155953	−4.09421	5.00E‐05	0.00040997	XLOC_018783	–	chr1	0.454788	3.90208	3.10098	5.00E‐05	0.00040997	XLOC_001370
Lpl	chr16	12.7953	0.820023	−3.96381	5.00E‐05	0.00040997	XLOC_008294	Clstn2	chr8	0.172923	1.28356	2.89195	0.0004	0.00258675	XLOC_019864
–	chr7	1.99245	0.132793	−4.01647	0.0002	0.0014157	XLOC_018784	Mag	chr1	10.9565	74.1972	2.75958	5.00E‐05	0.00040997	XLOC_001722

Gene	Locus	FPKM_Tpm4.2	FPKM_WT	Fold change	*P*_value	*q*_value	Gene_id	Gene	Locus	FPKM_Tpm1.12	FPKM_WT	Fold change	*P*_value	*q*_value	Gene_id
Tpm4.2 undiff. top 10 downregulated								Tpm4.2 undiff. Top 10 up‐regulated							
Dusp27	chr13	4.9844	0.451684	−3.46403	5.00E‐05	0.00040997	XLOC_006430	Foxa2	chr3	0.139538	5.6427	5.33765	0.00205	0.0102548	XLOC_012735
Nefl	chr15	18.1987	1.67599	−3.44074	5.00E‐05	0.00040997	XLOC_007467	–	chr5	0.0987955	3.14325	4.99167	0.0001	0.00076825	XLOC_016486
Pcdh20	chr15:	1.32061	0.133191	−3.30964	5.00E‐05	0.00040997	XLOC_007835	Pdpn	chr5	0.476489	13.7824	4.85424	5.00E‐05	0.00040997	XLOC_016368
–	chr12:	4.14833	0.470832	−3.13925	0.00465	0.020115	XLOC_005870	–	chr12	0.178894	4.40403	4.62165	0.0026	0.0124381	XLOC_005595
–	chr19	3.87688	0.456691	−3.08561	5.00E‐05	0.00040997	XLOC_010104	Qprt	*chr1*	0.281843	6.36273	4.49668	0.00065	0.00391153	XLOC_002237
Ptpn7	chr13	12.2785	1.481	−3.05149	5.00E‐05	0.00040997	XLOC_006040	Igf2 bp1	chr10	0.194826	3.9438	4.33933	5.00E‐05	0.00040997	XLOC_004304
–	chr5	6.19394	0.755452	−3.03545	0.01325	0.0466401	XLOC_016457	Bnc1	chr1	0.454089	6.59516	3.86036	5.00E‐05	0.00040997	XLOC_001936
Chrna7	chr1	1.76376	0.221772	−2.9915	0.00385	0.0172417	XLOC_001872	–	chrX	0.762958	10.7143	3.81179	5.00E‐05	0.00040997	XLOC_021139
–	chr16	8.87033	1.13533	−2.96588	0.0001	0.00076825	XLOC_008471	Spon1	chr1	0.289794	3.44618	3.5719	5.00E‐05	0.00040997	XLOC_000788
–	chr7	4.60091	0.598227	−2.94315	0.0114	0.0413927	XLOC_018791	Spp1	chr14	0.153611	1.66596	3.439	0.0056	0.0233655	XLOC_006940

### Differentiated Tpm1.12‐, Tpm3.1‐, and Tpm4.2‐overexpressing B35 cells exhibit upregulation of similar genes

*Arhgap25* was among the top upregulated genes in differentiated Tpm1.12‐, Tpm3.1‐, and Tpm4.2‐overexpressing cells, but not in Tpm2.1‐overexpressing cells (Table 2). Interestingly, *Arhgap25* encodes negative regulators of Rho‐GTPases, which are involved in actin remodeling, cell polarity, and migration 26. The upregulation of *Arhgap25*, only in response to cellular differentiation, suggests that in the differentiated Tpm1.12, Tpm3.1, and Tpm4.2 cells, Rho‐GTPases such as Rac or Cdc42 need to be silenced in order to restrict lamellipodia and filopodia formation 27. By contrast, differentiation of Tpm2.1 cells resulted in increases in genes that encode for proteins, such as *Kcnn4* and *Qprt*, which alter cell membrane polarization (Table 2).

**Table 2:** Top 10 up‐ and downregulated genes in differentiated B35 neuroblastoma cells overexpressing tropomyosin isoforms Tpm1.12, Tpm2.1, Tpm3.1, and Tpm4.2

Gene	Locus	FPKM_Tpm1.12	FPKM_WT	Fold change	*P*_value	*q*_value	Gene_id	Gene	Locus	FPKM_Tpm1.12	FPKM_WT	Fold change	*P*_value	*q*_value	Gene_id
Tpm1.12 diff. top 10 downregulated								Tpm1.12 diff. top 10 up‐regulated							
Il1rl1	chr9	1.59718	0.0488129	−5.03212	0.0001	0.0130135	XLOC_021158	Arhgap25	chr4	0.102724	3.17721	4.95092	5.00E‐05	0.00745967	XLOC_015519
Krt42	chr10	9.03038	0.286724	−4.97705	0.00035	0.034033	XLOC_004469	Cyp3a62	chr12	1.88881	37.393	4.30722	5.00E‐05	0.00745967	XLOC_005540
Myod1	chr1	9.5611	0.483197	−4.30649	5.00E‐05	0.00745967	XLOC_000524	–	chr1	0.137797	2.2002	3.99702	5.00E‐05	0.00745967	XLOC_000041
Tpm1	chr8	1512.31	88.6023	−4.09327	5.00E‐05	0.00745967	XLOC_020658	–	chr20	0.272328	3.87344	3.8302	0.0005	0.0437861	XLOC_012272
Hmx3	chr1	6.37178	0.391157	−4.02588	5.00E‐05	0.00745967	XLOC_000928	Rab15	chr6	0.182536	2.5494	3.80391	5.00E‐05	0.00745967	XLOC_017959
Lox	chr18	5.82529	0.36726	−3.98746	5.00E‐05	0.00745967	XLOC_009836	Adh1	chr2	0.344197	3.43627	3.31954	0.0002	0.022638	XLOC_011172
Wnt7b	chr7	6.69696	0.428724	−3.96538	5.00E‐05	0.00745967	XLOC_019352	Kcnn4	chr1	0.478335	3.63281	2.925	5.00E‐05	0.00745967	XLOC_000329
RGD1563159	chr18	2.89636	0.255698	−3.50173	0.0006	0.0495171	XLOC_009800	Car8	chr5	0.839724	6.01935	2.84162	0.0001	0.0130135	XLOC_016561
–	chr4	58.7075	5.6362	−3.38075	5.00E‐05	0.00745967	XLOC_015812	Col2a1	chr7	2.69563	17.742	2.71847	5.00E‐05	0.00745967	XLOC_019400
Kit	chr14	1.55982	0.155265	−3.32858	5.00E‐05	0.00745967	XLOC_007261	Mmp2	chr19	18.7413	112.613	2.58709	5.00E‐05	0.00745967	XLOC_010294

Gene	Locus	FPKM_Tpm2.1	FPKM_WT	Fold change	*P*_value	*q*_value	Gene_id	Gene	Locus	FPKM_Tpm2.1	FPKM_WT	Fold change	*P*_value	*q*_value	Gene_id
Tpm2.1 diff. top 10 downregulated								Tpm2.1 diff. top 10 up‐regulated							
MGC114427	chrX	20.2284	1.27503	−3.98777	5.00E‐05	0.00745967	XLOC_022195	Cyp3a62	chr12	0.798617	37.393	5.54912	5.00E‐05	0.00745967	XLOC_005540
Hs6st2	chrX	1.22422	0.0811244	−3.91558	5.00E‐05	0.00745967	XLOC_022531	–	chr20	0.322848	3.87344	3.58469	0.0005	0.0437861	XLOC_012272
RGD1563159	chr18	2.95816	0.255698	−3.53219	0.00055	0.046874	XLOC_009800	Kcnn4	chr1	0.326822	3.63281	3.47451	5.00E‐05	0.00745967	XLOC_000329
–	chr12	10.0635	1.02525	−3.29509	0.0003	0.0304187	XLOC_005835	Qprt	chr1	0.599728	6.25604	3.38287	5.00E‐05	0.00745967	XLOC_002288
Enpp2	chr7	1.5044	0.162351	−3.212	0.0006	0.0495171	XLOC_019171	Col2a1	chr7	1.90447	17.742	3.21971	5.00E‐05	0.00745967	XLOC_019400
–	chr14	4.84023	0.632595	−2.93572	5.00E‐05	0.00745967	XLOC_006938	Slc27a3	chr2	0.209313	1.69112	3.01425	5.00E‐05	0.00745967	XLOC_011597
RGD1562638	chr16	2.90249	0.385049	−2.91418	5.00E‐05	0.00745967	XLOC_008380	Pcsk9	chr5	0.295631	2.07142	2.80875	5.00E‐05	0.00745967	XLOC_016796
–	chr12	1.6546	0.223779	−2.88634	0.0003	0.0304187	XLOC_005779	Magea11	chrX	2.2022	14.4629	2.71534	5.00E‐05	0.00745967	XLOC_022188
LOC100302465	chr1	20.4765	3.02484	−2.75904	5.00E‐05	0.00745967	XLOC_002336	–	chr5	0.820127	5.38397	2.71475	5.00E‐05	0.00745967	XLOC_016535
Hmx3	chr1	2.58082	0.391157	−2.72201	5.00E‐05	0.00745967	XLOC_000928	Capn6	chrX	0.426284	2.42179	2.50619	0.00025	0.0266766	XLOC_022475

Gene	Locus	FPKM_Tpm3.1	FPKM_WT	Fold change	*P*_value	*q*_value	Gene_id	Gene	Locus	FPKM_Tpm3.1	FPKM_WT	Fold change	*P*_value	*q*_value	Gene_id
Tpm3.1 diff. top 10 downregulated								Tpm3.1 diff. top 10 up‐regulated							
Enpp2	chr7	24.6796	0.162351	−7.24806	0.0001	0.0130135	XLOC_019171	Cyp3a62	chr12	1.04867	37.393	5.15614	5.00E‐05	0.00745967	XLOC_005540
Prrx1	chr13	4.94714	0.0731377	−6.07983	0.0002	0.022638	XLOC_006645	Arhgap25	chr4	0.296596	3.17721	3.42119	5.00E‐05	0.00745967	XLOC_015519
Col3a1	chr9	23.4538	0.808091	−4.85916	5.00E‐05	0.00745967	XLOC_021174	Rasal3	chr7	0.92198	7.6725	3.05689	5.00E‐05	0.00745967	XLOC_018972
–	chr16	14.9788	0.625436	−4.58191	5.00E‐05	0.00745967	XLOC_008386	Slc30a3	chr6	2.96113	16.6391	2.49036	5.00E‐05	0.00745967	XLOC_017347
Wnt16	chr4	3.20669	0.135369	−4.56612	5.00E‐05	0.00745967	XLOC_014664	Nkx2‐8	chr6	3.62869	20.149	2.47319	5.00E‐05	0.00745967	XLOC_017909
–	chr8	1.72658	0.112733	−3.93694	5.00E‐05	0.00745967	XLOC_020613	Rgs16	chr13	0.719619	3.38166	2.23243	0.00015	0.0182456	XLOC_006309
Hs6st2	chrX	1.16506	0.0811244	−3.84413	5.00E‐05	0.00745967	XLOC_022531	–	chr2	4.50913	20.1249	2.15806	0.00055	0.046874	XLOC_011019
–	chr19	3.2643	0.247639	−3.72046	5.00E‐05	0.00745967	XLOC_010230	Hsd11b1	chr13	0.880275	3.73038	2.0833	0.0006	0.0495171	XLOC_006786
Slc14a1	chr18	27.2188	2.33361	−3.54397	5.00E‐05	0.00745967	XLOC_009903	Mcoln3	chr2	0.771953	3.15482	2.03097	5.00E‐05	0.00745967	XLOC_011198
Wnt5a	chr16	1.1231	0.0965497	−3.54007	0.00035	0.034033	XLOC_008210	Cryab	chr8	11.7217	46.2096	1.97902	5.00E‐05	0.00745967	XLOC_019966

Gene	Locus	FPKM_Tpm4.2	FPKM_WT	Fold change	*P*_value	*q*_value	Gene_id	Gene	Locus	FPKM_Tpm4.2	FPKM_WT	Fold change	*P*_value	*q*_value	Gene_id
Tpm4.2 diff. top 10 downregulated								Tpm4.2 diff. top 10 up‐regulated							
Myod1	chr1	28.5968	0.483197	−5.8871	5.00E‐05	0.00745967	XLOC_000524	Spint2	chr1	0.423831	4.9636	3.54983	0.00035	0.034033	XLOC_001727
Car3	chr2	28.0891	1.45161	−4.27428	5.00E‐05	0.00745967	XLOC_011390	Col2a1	chr7	1.57159	17.742	3.49687	5.00E‐05	0.00745967	XLOC_019400
Krt15	chr10	303.929	17.8834	−4.08704	5.00E‐05	0.00745967	XLOC_004463	Arhgap25	chr4:	0.482218	3.17721	2.72	5.00E‐05	0.00745967	XLOC_015519
Med12l	chr2	3.3854	0.213251	−3.9887	5.00E‐05	0.00745967	XLOC_010857	Kcnn4	chr1	0.678271	3.63281	2.42115	5.00E‐05	0.00745967	XLOC_000329
Atp1a3	chr1	4.88898	0.450264	−3.44069	5.00E‐05	0.00745967	XLOC_001683	Calcb	chr1	3.10533	16.2157	2.38457	5.00E‐05	0.00745967	XLOC_000822
Efna5	chr9	1.64816	0.152239	−3.43644	0.00035	0.034033	XLOC_021706	–	chrX	1.53544	7.26066	2.24145	5.00E‐05	0.00745967	XLOC_022048
–	chr2	15.2347	1.40862	−3.435	0.0001	0.0130135	XLOC_011936	–	chrX	1.46876	6.94188	2.24073	5.00E‐05	0.00745967	XLOC_022391
Chrna1	chr3	183.471	18.6364	−3.29935	5.00E‐05	0.00745967	XLOC_013760	–	chrX	0.596817	2.79741	2.22873	0.00045	0.0405794	XLOC_022361
Ass1	chr3	1.98046	0.211055	−3.23015	0.0001	0.0130135	XLOC_013634	Pcdh7	chr14	0.778927	3.15898	2.0199	0.0001	0.0130135	XLOC_007301
Trim55	chr2	3.14264	0.345104	−3.18687	0.00035	0.034033	XLOC_010770	Shtn1	chr1	0.693723	2.43258	1.81005	0.0004	0.0375237	XLOC_002745

### DEGs arising from the overexpression of tropomyosin isoforms cluster into various pathways

In undifferentiated cells, cluster analysis of DEGs from the overexpression of the four tropomyosin isoforms, used in this study, results in the emergence of multiple pathways. In differentiated cells, Tpm3.1 is the only isoform where DEGs cluster into pathways.

#### Tpm1.12

**Figure 2:**
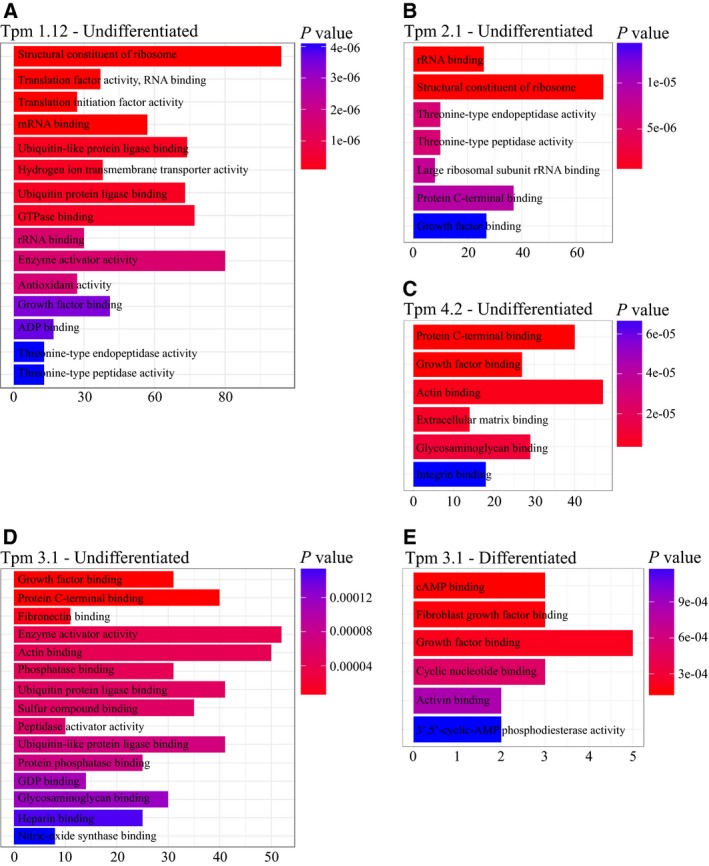
Pathway analysis of DEGs from undifferentiated and differentiated B35 cells overexpressing Tpm isoforms. (A–D) Overexpression of Tpm1.12, Tpm2.1, Tpm3.1, and Tpm4.2 in undifferentiated B35 cells generates DEGs that cluster into various GO terms. (E) GO terms generated from the clustering of DEGs from Tpm3.1 overexpression in differentiated B35 cells.

The DEGs arising from Tpm1.12 overexpression in undifferentiated cells cluster into 15 GO terms (Fig. 2A). Of these GO terms, ribosomal, RNA, and ubiquitin related are among the pathways holding the highest statistical significance. Ribosomal proteins are most commonly known to be involved in protein synthesis but have also been shown to exert extraribosomal functions including immune signaling and development of various cell types 28 as well as being implicated in various cancers including glioblastoma, gastrointestinal, prostate, and lung 29, 30, 31, 32. In this study, expression levels of many genes, encoding ribosomal proteins (Rp), were found to be differentially regulated in both Tpm1.12‐ and Tpm2.1‐overexpressing cells including *Rpl9, Rpl13, Rpl19,* and *Rpl22*.

In the GTPase binding GO term, the *Pfn1* (profilin 1) is found to be upregulated. Pfn1 is involved in actin nucleation, mediating the exchange of ADP to ATP on monomeric actin. This process primes the actin monomer to be incorporated into the growing ‘barbed end’ of actin filaments, resulting in actin filament polymerization 33. Overexpression of Tpm1.12 has previously been shown to promote neurite branching and filopodia formation 13, two processes that rely on polymerization of actin filaments. The upregulation of *Pfn1* is a potential mechanism by which Tpm1.12 is able to enhance neurite branching and filopodia formation in the cell.

#### Tpm2.1

Differentially expressed genes from undifferentiated cells overexpressing Tpm2.1 cluster into 7 GO terms (Fig. 2B), rRNA binding, structural constituent of ribosome, threonine‐type endopeptidase activity, threonine‐type peptidase activity, large ribosomal subunit rRNA binding, protein C‐terminal binding, and growth factor binding. Within the protein C‐terminal‐binding protein pathway are genes, associated with apoptosis. Tpm2.1 is known to have tumor‐suppressing properties in breast and urinary bladder cancers 10, 34, 35 and has recently been shown to increase cell sensitivity to apoptosis by detachment from the extracellular matrix, referred to as anoikis, and through the modulation of various apoptosis‐inducing proteins 17.

In this study, the overexpression of Tpm2.1 shows upregulation of *Dapk3* (death‐associated protein kinase 3) and downregulation of *Cdc37* (cell division cycle 37) gene expression levels. These two genes are involved in apoptosis and clustered into the protein C‐terminal GO term (Fig. 2B). The Dapk family are actin cytoskeleton‐associated Ca2 + /calmodulin (CaM)‐regulated serine/threonine kinases reported to regulate cell death via various mechanisms including interferon‐γ, c‐Myc, and anoikis 36, 37, 38, 39. Dapk3 has been shown to exert apoptotic function through mitochondrial pathways 40 and to have tumor‐suppressing qualities 38, 41, 42.

Cdc37 is a cochaperone protein to heat‐shock protein 90 (HSP90). Cdc37 facilitates the interaction of protein kinases with HSP90 by arresting the ATPase cycle of HSP90 and inducing an open conformational state that promotes client protein interaction 43. HSP90 in collaboration with Cdc37 has been suggested to promote the proliferation and survival of cancer cells through dysregulation of oncogenes 44, and silencing Cdc37 has been shown to enhance cell cycle arrest and apoptosis 45, 46, 47.

In our study, Tpm2.1 overexpression results in the upregulation of *Dapk3* and the downregulation of *Cdc37* genes. The ability of Tpm2.1 to modulate the expression of these genes may help to shed light onto the tumor‐suppressing and proapoptotic characteristics of Tpm2.1.

#### Tpm4.2

Differentially expressed genes that were observed in response to the overexpression of Tpm4.2 cluster into six GO terms, protein C‐terminal binding, growth factor binding, actin binding, extracellular matrix binding, glycosaminoglycan binding, and integrin binding (Fig. 2C).

The integrin‐binding pathway includes genes for integrin subunits. Integrins function in cell surface adhesion and signaling, acting as a mediator between the intracellular actin cytoskeleton and the extracellular matrix 48. Gene expression of *Itga7* (Integrin subunit α7) is found to be upregulated in response to Tpm4.2 overexpression in undifferentiated B35 cells. Itga7 and β‐subunits form heterodimeric integrin receptors that bind laminin and regulate cell adhesion 49. Interestingly, Itga7 has been found to play a role in lamellipodia formation 50, neuritogenesis of cortical neurons 51, and the regeneration of subpopulations of injured sensory neurons 52. In a previous study by Curthoys *et al*. 13, overexpression of Tpm4.2 in undifferentiated B35 neuroblastoma cells led to an increase in neurite branching, filopodia formation, and growth cone size. It is plausible that this observed phenotype may be promoted by the ability of Tpm4.2 to modulate genetic expression of genes such as Itga7. Furthermore, overexpression of Tpm4.2 increases protein levels of fascin 13, an actin‐binding protein recruited to actin bundles during filopodia and lamellipodia formation 53.

#### Tpm3.1

Gene ontology terms arising from overexpression of Tpm3.1 in undifferentiated cells include growth factor‐binding, protein C‐terminal‐binding, fibronectin‐binding, and actin‐binding pathways among others (Fig. 2D).

The protein C‐terminal‐binding and fibronectin‐binding pathways comprise genes involved in cell adhesion, migration, and motility including *Fbln5* (fibulin‐5) and *Itga4* (integrin subunit gene α4).

Fbln5 is an extracellular matrix protein involved cell adhesion. In endothelial cells, Fbln5 has been shown to operate via integrin binding 54 enhancing cell attachment and adhesion as well as decreasing proliferation 55. Interestingly, Fbln5 is reported to have context‐dependent oncogenic and tumor‐suppressing roles 56. Increased levels of Fbln5 reduce cell migration and invasion in ovarian and breast cancer 57, 58.

Itga4 and β1‐subunits form integrin heterodimers that bind fibronectin, increasing focal adhesion and cell motility 59. Furthermore, the cytoplasmic tail of α4 integrins binds actin filament‐bound nonmuscle myosin IIa to regulate cell migration 60.

In the current study, overexpression of Tpm3.1 upregulates the expression levels of both *Fbnl5* and *Itga4*. Previously, Tpm3.1 has been shown to recruit myosin IIa into stress fibers, stabilize actin filaments, and slow cell migration 9, 14, 19. It is plausible then that Tpm3.1 works in conjunction with Fbln5, Itga4, and myosin IIa to enhance cell stability and adhesion.

#### Tpm3.1 overexpression in differentiated B35 cells

Interestingly, in differentiated cells, Tpm3.1 was the only isoform that generated GO terms from cluster analysis of DEGs (Fig. 2E). The GO terms observed include growth factor‐binding, glycosaminoglycan‐binding, water transmembrane transporter activity, RNA polymerase II transcription coactivator activity GO terms (Fig. 2E). The greatest difference was observed in the GO terms growth factor binding and glycosaminoglycan binding. Differentiated Tpm3.1 cells have increased levels of fibroblast growth factor receptors 1 and 2 (*Fgfr1* and *Fgfr2*, respectively). Increased expression of Fgfr1 is associated with parathyroid carcinoma 61. Tpm3.1 is the predominant Tpm isoform in numerous cancers 7. Taken together, Fgfr1 and Tpm3.1 could be working synergistically during cancer development. Furthermore, Fgfr2 expression is upregulated in differentiating mouse podocytes *in vitro*, due to the reorganization of the actin cytoskeleton and extension of their cellular processes 62. Therefore, as B35 cells overexpressing Tpm3.1 undergo differentiation, they may require increased levels of Fgfr1 and Fgfr2 to accommodate the reorganization of the actin cytoskeleton.

### Actin‐binding pathway

Undifferentiated B35 cells overexpressing either Tpm3.1 or Tpm4.2 are found to differentially regulate genes that cluster into the actin‐binding GO term (Table 3). Both Tpm3.1‐ and Tpm4.2‐overexpressing B35 cells show altered genetic read levels of members of the Coronin family. In particular, *Coro1a* is upregulated in both Tpm3.1‐ and Tpm4.2‐overexpressing cells. The role of the Coronin family has been well described in immune cells, with recent evidence also suggesting a role in neuronal cells 63. Interestingly, CORO1A is associated with polarized cells, suggesting that it is required for active actin cytoskeleton rearrangement and protein synthesis 63. Furthermore, members of the type 1 Coronin family typically localize to protrusions of cell membranes, where they modulate actin dynamics 64. Therefore, the increased neurite outgrowth and axonal extension known to be associated with increased Tpm3.1 expression 65, 66, 67 may be assisted by increased *Coro1a* levels. Further investigations are needed to elucidate the interaction between Coro1a and Tpm3.1/Tpm4.2.

**Table 3:** Overexpression of tropomyosin isoforms Tpm1.12, Tpm3.1, and Tpm4.2 differentially regulates the expression of genes involved in actin binding

Gene	Locus	FPKM_Tpm3.1	FPKM_WT	Fold change	*P*_value	*q*_value	Gene_id
Tpm3.1 undiff. DEGs in actin‐binding pathway							
Coro1a	chr1	33.3272	19.2652	−0.790702	5.00E‐05	0.000409973	XLOC_002225
Fmnl1	chr10	1.16689	2.0026	0.779204	0.00185	0.00941884	XLOC_003627
Myh10	chr10	6.3366	10.0299	0.66253	5.00E‐05	0.000409973	XLOC_003283
Myo7a	chr1	17.4539	9.04809	−0.94786	5.00E‐05	0.000409973	XLOC_001985
Limch1	chr14	12.9913	20.9554	0.689775	5.00E‐05	0.000409973	XLOC_007048
Twf2	chr8	14.5084	21.8628	0.591586	0.0001	0.000768253	XLOC_019311

Gene	Locus	FPKM_Tpm4.2	FPKM_WT	Fold change	*P*_value	*q*_value	Gene_id
Tpm4.2 undiff. DEGs in actin‐binding pathway							
Marcksl1	chr1	20.8434	28.8606	0.469513	0.00785	0.0307347	XLOC_001671
Coro1a	chr1	31.1256	19.2652	−0.692101	5.00E‐05	0.000409973	XLOC_002225
Myh10	chr10	3.76493	10.0299	1.41362	5.00E‐05	0.000409973	XLOC_003283
Myh1	chr10	58.7752	32.3774	−0.860222	5.00E‐05	0.000409973	XLOC_003272
Slc6a4	chr10	2.18532	6.9866	1.67674	5.00E‐05	0.000409973	XLOC_004178
Tmod2	chr8	2.31106	0.981818	−1.23503	0.00015	0.00110539	XLOC_019804

Gene	Locus	FPKM_Tpm1.12	FPKM_WT	Fold change	*P*_value	*q*_value	Gene_id
Tpm1.12 undiff. DEGs in actin‐binding pathway							
Marcksl1	chr1	0.456449	1.35277	1.56739	5.00E‐05	0.000409973	XLOC_001670
Coro1a	chr1	30.2034	19.2652	−0.648712	5.00E‐05	0.000409973	XLOC_002225

Despite their commonalities, Tpm3.1 overexpression and Tpm4.2 overexpression also result in differential changes in genes from the GO terms actin‐binding pathway. Whereas the overexpression of Tpm4.2 results in a decrease in *Marcksl1*, this is not observed for Tpm3.1. Marcksl1 is an actin cross‐linking protein, which undergoes phosphorylation to bundle and stabilize F‐actin 68. The inhibition of Marcksl1 phosphorylation causes an increase in actin mobility, compromised filopodia formation, enhanced lamellipodium formation, and cell migration 68. Therefore, the increase in filopodia number previously observed in Tpm4.2‐overexpressing cells 13 may be partly attributed to a concomitant decrease in *Marcksl1*.

Although the overexpression of Tpm1.12 did not lead to DEGs clustering to form an actin‐binding pathway, both *Coro1a* and *Marcksl1* genes are found to be differentially expressed. As observed in Tpm4.2 overexpression, Tpm1.12 overexpression upregulates *Coro1a* and downregulates *Marcksl1* (Table 3). The similar changes, induced by Tpm4.2 and Tpm1.12, are consistent with the study by Curthoys *et al*. 13, where overexpression of Tpm1.12 was found to have similar effects on cell morphology as Tpm4.2, with an increase in filopodia and neurite branching.

### Potential mechanisms for transcriptional changes caused by Tpm expression

Our data demonstrate that Tpm expression leads to isoform‐dependent transcriptional changes in eukaryotic cells. The mechanisms by which Tpms lead to these transcriptional changes are still unknown. A role for actin in transcriptional regulation has been well established and reviewed previously 69, 70. A potential mechanism of transcriptional regulation by Tpms may be via Tpm isoform‐dependent regulation of actin turnover which is important for the localization of transcriptional regulators. Maintaining the balance between the globular (G) and the filamentous (F) pool of cytoplasmic actin has been implicated in the translocation several transcriptional regulators, including the homeobox transcription factor PREP2 71, the transcriptional repressor YY1 72, and the transcriptional coactivator MAL 73. Regulation of the cytoplasmic pool of G‐ and F‐actin by the expression levels of different Tpms could therefore impact the transcriptome as a consequence of altered translocation of these transcriptional regulators. Tpms have also been found in the nucleus 74, 75, where they may directly regulate transcription of the genes that group in various pathways identified in our study.

## Conclusion

In conclusion, overexpression of Tpm isoforms in undifferentiated B35 neuroblastoma cells leads to the differential expression of a plethora of genes. However, once differentiated, Tpm isoforms have a weaker influence on gene expression. In undifferentiated cells, DEGs cluster into various pathways that show some similarity between isoforms. In differentiated cells, the overexpression of Tpm3.1 was the only isoform to generate GO terms. Many of the observed DEGs are involved in cellular activities that relate to Tpm functions, suggesting that Tpms can modulate cell dynamics and properties by regulating specific genes. Overall, this study highlights the ability of Tpm isoforms to regulate patterns of gene expression in an isoform‐specific manner and aligns with their capacity to similarly control actin filament function.

## Author contributions

TF and MJ designed the study. HS, AKS, and BJC carried out the experiments and analyzed the data. HS, AKS, and TF wrote the manuscript. MJ and PWG helped in editing the manuscript.
